# A machine learning-based depression risk prediction model for healthy middle-aged and older adult people based on data from the China health and aging tracking study

**DOI:** 10.3389/fpubh.2025.1515094

**Published:** 2025-08-06

**Authors:** Fang Xia, Jie Ren, Linlin Liu, Yanyin Cui, Yufang He

**Affiliations:** ^1^School of Health Management, Changchun University of Traditional Chinese Medicine, Changchun, China; ^2^School of Humanities and Management, Zhejiang Chinese Medical University, Hangzhou, China

**Keywords:** prediction model, depression, machine learning, older adults, China longitudinal study

## Abstract

**Background:**

Predicting depression risk in adults is critical for timely interventions to improve quality of life. To develop a scientific basis for depression prevention, machine learning models based on longitudinal data that can assess depression risk are necessary.

**Methods:**

Data from 2,331 healthy older adults who participated in the China Health and Retirement Longitudinal Study (CHARLS) from 2018 to 2020 were used to develop and validate the predictive model. Depression was assessed using the 10-item Center for Epidemiologic Studies Depression Scale (CES-D-10), with a score of ≥10 indicating depressive symptoms. Several machine learning algorithms, including logistic regression, k-nearest neighbor, support vector machine, multilayer perceptron, decision tree, and XGBoost, were employed to predict the 2-year depression risk. The dataset was randomly split into a training set (70%) and a testing set (30%), and hyperparameters were optimized in the training phase. The models’ performance was evaluated in the testing set using accuracy, sensitivity, specificity, area under the receiver operator characteristic (ROC) curve, and F1 score. Model interpretability was enhanced using SHapley Additive exPlanations (SHAP).

**Results:**

A total of 563 (24.15%) participants developed depression during the 2-year follow-up period. LASSO regression identified 12 key predictive features from an initial set of 26. Among the six models tested, XGBoost exhibited the best predictive performance, achieving the highest area under the ROC curve (0.774), accuracy (0.722), sensitivity (0.757), and F1 score (0.720), with a specificity of 0.687. Decision curve analysis (DCA) confirmed the net clinical benefit of the XGBoost model across most threshold ranges. SHAP interpretation revealed that cognitive ability, total income, life satisfaction, sleep quality, and pain were the top five most influential factors in predicting depression risk.

**Conclusion:**

Our findings support the feasibility of using machine learning-based models to predict depression risk in healthy older adults over a 2-year period. The integration of XGBoost and SHAP enhances model interpretability, offering valuable insights into individual risk factors. This approach enables personalized risk assessment, which may help develop targeted interventions for depression prevention in aging populations.

## 1 Introduction

People aged 45 and over account for a significant proportion of the global population, and this number is expected to increase by 2050 (1). As they age, middle-aged and older adults face various health problems, with depression being particularly prevalent. The global prevalence of depression among middle-aged and older adults has been estimated to range from 10 to 20%, with some studies reporting even higher rates, especially in regions like China where the aging population is growing rapidly. Not only does this mental health problem affect the quality of life, but it is also associated with adverse health outcomes including worsening of chronic diseases, increased hospitalization, and higher mortality (2–5). Thus, extending the healthy lifespan and independence of middle-aged and older adults has become an important goal for society (6, 7). Various factors associated with depression among middle-aged and older adults have been identified, including chronic illness, cognitive decline, social isolation, financial difficulties, and life events (8–12). In addition to increasing the risk of depression, these factors may exacerbate other health problems, creating a vicious cycle (13). As mental health problems tend to be neglected among middle-aged and older adults, they may lead to deterioration of physical health. For example, depression is strongly associated with poor management of chronic conditions, such as cardiovascular disease and diabetes (14–17). Therefore, accurate prediction of depression risk and targeted interventions are essential to reduce the burden of depression in middle-aged and older adults. Given the significant prevalence of depression, especially in older populations, understanding its risk factors and potential interventions is crucial to improving both mental and physical health outcomes for this group. To achieve this goal, advanced predictive modelling and data analysis techniques are required. Machine learning methods have shown superiority in medical predictive models used to identify high-risk groups for depression, supporting the development of effective prevention strategies (18–20).

Machine learning methods can identify non-linear relationships and ostensibly irrelevant factors that are difficult to detect with traditional methods, leading to more accurate feature selection (21, 22). In this study, we use a variety of machine learning algorithms, including logistic regression, KNN, multilayer perceptron (MLP), decision trees, support vector machines, and XGBoost, to develop and evaluate predictive models for depression. It should be noted that many previous machine learning modelling studies have encountered problems (23, 24). To build more accurate and generalizable models, this study used LASSO regression for feature selection, resampling techniques to address category imbalance, and normalization of training and test data. Hyperparameter tuning was performed using grid search to improve model performance, while DeLong testing was used to prevent overfitting. We then compared the performance of various machine learning algorithms based on performance metrics, including area under the receiver operator characteristic (ROC) curve (AUC), accuracy, sensitivity, specificity, and F1 score. Due to the complex, non-linear relationships identified by some machine learning algorithms, the results are often difficult to interpret and result in the “black box” problem, which limits the practical applications of predictive models (25). To overcome this issue, we apply SHapley Additive exPlanations (SHAP) to the best performing models to interpret individual predictions from kernel-based and tree-based models. SHAP offers significant advantages over other interpretation methods for visualizing complex machine learning prediction models (26), helping address the “black box” problem. Notably, this advanced model interpretation method has not yet been used to predict the risk of depression in healthy Chinese older adults.

Overall, this study aimed to develop and validate a model to predict the two-year incidence of depression in healthy Chinese older adults using six machine learning algorithms. These findings can be used to help improve the mental health of middle-aged and older adults, enhance their quality of life through individualized interventions, reduce the burden on the healthcare system, and provide new directions for future research.

## 2 Materials and methods

### 2.1 Data and participants

Data used for model development were obtained from the China Health and Retirement Tracking Survey (CHARLS) (27), a longitudinal survey of Chinese residents aged 45 years and older. CHARLS is harmonized with leading international studies in the Health and Retirement Study (HRS) model, ensuring best practices and comparability with similar international surveys. A stratified (by GDP per capita in urban and rural counties), multi-stage (county/district, village/community, household), PPS random sampling strategy was used. The baseline CHARLS survey was initiated in 2011–2012, with second to fifth waves of follow-up surveys in 2013, 2015, 2018, and 2020. Given the extensive use and validation of the CHARLS dataset in related literature, and its alignment with internationally recognized standards, no additional reliability and validity testing was performed for this study. To ensure the sample’s representativeness of the national population, the baseline survey covered 150 counties/districts and 450 villages/urban communities, including 10,257 households and 17,708 respondents, thus reflecting the overall situation of China’s middle-aged and older adult population.

To model depression over a 2-year period, we obtained data for 20,180 participants in the 2018 and 2020 study waves. The inclusion criteria were: (1) participants aged 45 years or older, (2) participants who completed the Central Depressive Symptom Inventory (CES-D 10) in the 2018 and 2020 surveys, and (3) participants with a CES-D 10 score <10 in the 2018 survey, indicating no depressive symptoms at baseline. Exclusion criteria were: (1) participants with incomplete or missing data, (2) participants who were unable to complete the questionnaire independently due to cognitive impairment or other health problems, and (3) participants who were unable to complete the follow-up visit during the baseline survey due to personal reasons or health problems. Ultimately, 2,331 participants met these criteria and were included in model development and internal validation.

### 2.2 Research variables

#### 2.2.1 Outcome variables

Depression was assessed using the CES-D 10 (28, 29), which consists of 10 items designed to assess depressive symptoms over the past week. Each item is rated on a scale from 0 to 3, with 0 indicating “little or no days (< 1 day),” 1 indicating “some days (1–2 days),” 2 indicating “occasional or moderate days (3–4 days),” 3 indicating “occasional or moderate days (3–4 days),” and 4 indicating “no time (less than a day).” “, and 3 means “most or all of the time (5–7 days).” Total scores range from 0 to 30, with higher scores, indicating more severe depressive symptoms. Participants with a CES-D 10 score < 10 were categorized as having no depressive symptoms, while those scoring 10 or higher were categorized as having depressive symptoms (30).

#### 2.2.2 Predictor variables

We conducted a preliminary assessment of predictors associated with depression based on their clinical significance, scientific knowledge, and predictive models developed in previous studies (31, 32). Based on this assessment, we selected 26 factors potentially associated with depression including demographic characteristics (age, gender, marital status, education); lifestyle and health behaviors (life satisfaction, smoking, alcohol consumption, sleep duration, social activities, exercise, cognitive functioning); health status (incapacitation, body pain, disability, falls, hip injury, exercise level, high blood pressure); economic status (total household income, percentage of per capita household income, receipt of pension, parental financial support for children); family structure (family size, number of living children); and other factors (work-retirement status).

#### 2.2.3 Data collection

Participants’ demographic characteristics, lifestyle and health behaviors, and health status were collected by trained staff using questionnaires. Disability was assessed through activities of daily living (ADLs) and instrumental activities of daily living (IADLs). ADLs included basic tasks such as dressing, bathing, eating, waking up, using the toilet, and controlling defecation. IADLs involved complex tasks such as performing household chores, preparing a hot meal, shopping, managing money, making phone calls, and taking medication. Responses were divided into four categories: (1) no difficulty, (2) difficulty, but able to complete, (3) difficulty and need help, and (4) unable to complete. Each ADL/IADL item was scored 0 if the participant had no problems performing the activity, and 1 if they had difficulties or were unable to complete the task. The item scores were then summed and participants were grouped into two categories as following: (1) no loss of functioning (ADL/IADL score = 0) and (2) loss of functioning (ADL/IADL score ≥1). Life satisfaction was determined using a questionnaire with items about health, marriage, children, and overall life that were scored from 1 (very dissatisfied) to 5 (very satisfied). Data related to health behaviors were collected through self-report questionnaires and physical examinations and included physical pain, falls, hip injuries, hypertension, and level of exercise.

### 2.3 Statistical analyses

#### 2.3.1 Data pre-processing

For the dataset used in this study, the percentage of variables with missing values was extremely low (<0.15%). Therefore, the R package “mice” was employed to fill in missing values by multiple imputation using the regression model. Notably, 24.15% of all participants were disabled, which may have contributed to a decrease in classifier performance. To address this imbalance, SMOTE (Synthetic Minority Oversampling Technique) was applied, which oversamples the minority class by generating synthetic samples from linear combinations of existing minority class neighbors. The data were divided into training and test sets in a 7:3 ratio. Count data were presented as numerical values and proportions and analyzed using the chi-square test. For continuous data that did not follow a normal distribution, median and interquartile range were presented and analyzed using the Mann–Whitney U test. During the exclusion process, individuals with severe missing data or those who were unable to provide complete data due to reasons such as death, inability to contact, or unwillingness to participate were excluded. Excluded individuals may have systemic differences, particularly those who were older, in worse health, or had lower socioeconomic status, which might have led to their exclusion. While this could have a potential impact on the results, the exclusion criteria were strictly followed to ensure data quality and the reliability of the analysis. Data processing and analysis were performed using Stata and R software, with all statistical analyses conducted using R version 4.4.0. A significance level of *p* < 0.05 was used.

#### 2.3.2 Model construction and assessment

Prior to modelling, the training and test sets were normalized. The training set was used to construct the model and the test set was used to optimize the model parameters and evaluate its generalization ability. The steps used to construct and evaluate the model are as follows: (1) Feature selection: the least absolute shrinkage and selection operator (LASSO) regularization method was applied to the training set to identify significant features from the initial 26 variables. To improve reliability, the algorithm was cross-validated 10-fold. (2) Model construction: six different machine learning algorithms (including logistic regression (LR), k-nearest neighbors (KNN), decision tree (DT), multilayer perceptron (MLP), random forest (RF), and extreme gradient boosting (XGBoost)) were used to construct the model. Model optimization was performed through 10-fold cross-validation and 5 iterations, while hyperparameters were tuned using the grid search to ensure model stability. (3) Model evaluation: model performance was assessed by the area under the curve (AUC) of the subject operating characteristics (ROC), accuracy, sensitivity, specificity, and F1 score, while the Youden index was used to select the optimal threshold. Decision curve analysis (DCA) was also performed on the test set to assess the value of each model in practical applications.

#### 2.3.3 Interpretation of the model

**Figure 1:**
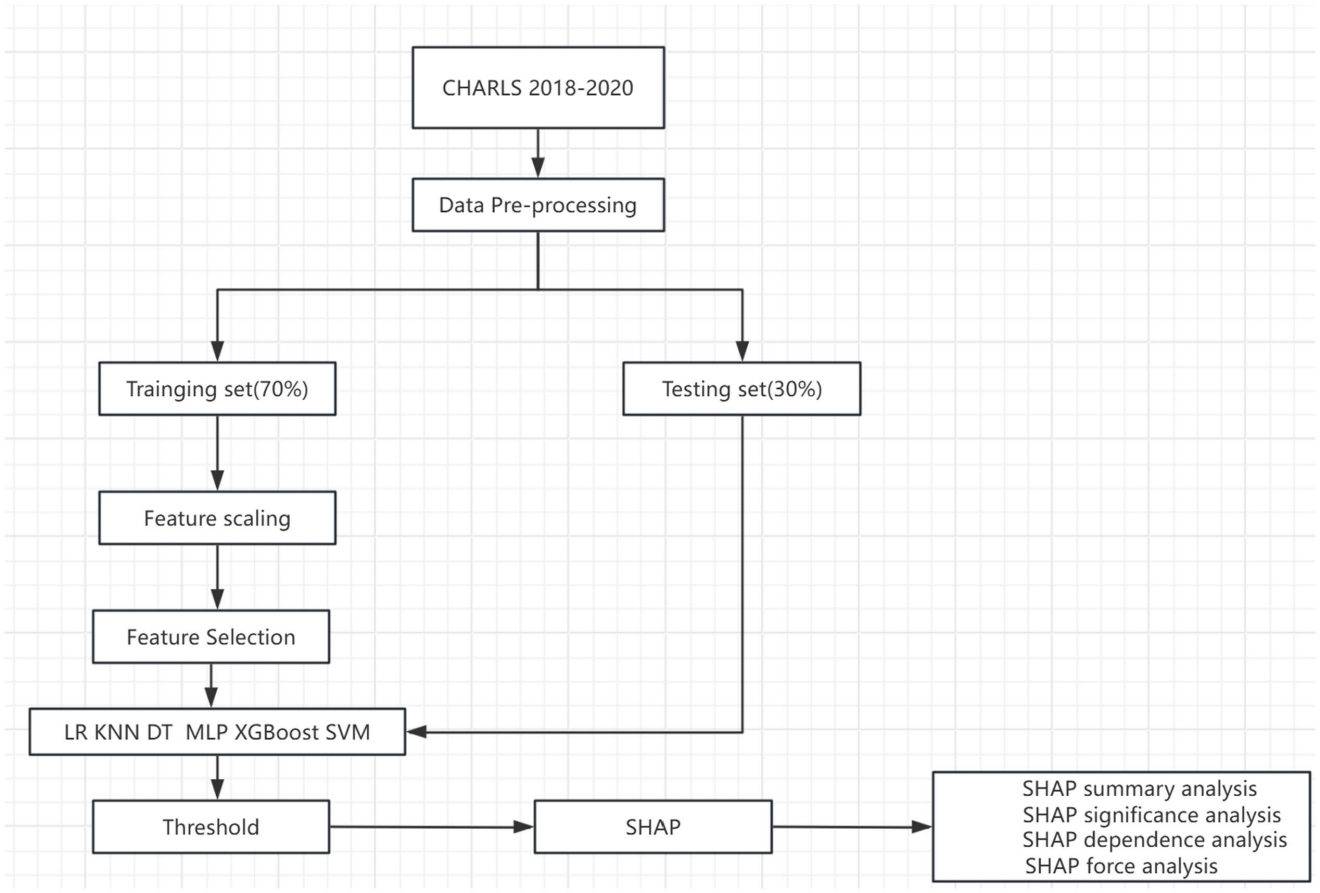
Flow chart.

Figure 1 shows the overall workflow of this study. Interpreting machine learning models is a known challenge. To help explain the impact of each feature variable on the final model, we employed the SHapley Additive eXplanation (SHAP) method. The SHAP value estimates the contribution of each feature to the predicted outcome based on game theory, treating each feature as a participant and fairly attributing to each feature, thus explaining its contribution to the individual prediction. We assessed the importance of each feature by calculating the average absolute value of its SHAP and plotted the feature SHAP values for each sample to understand the overall pattern and its range of influence on the dataset. The non-linear effects of the features were assessed using SHAP dependency plots. Two SHAP prediction examples are provided as demonstration.

## 3 Results

### 3.1 Data processing results

Of the 2,331 older adults initially in good health, after 2 years of follow-up, 563 had become depressed (24.15%). Due to the data imbalance between the depressed and non-depressed groups, after applying SMOTE, we were left with a sample of 3,457, of which 1,689 (48.86%) were identified as depressed. Data were split in a 7:3 ratio, with the training set containing 2,420 cases (1,183 depressed) and the test set containing 1,037 participants (506 depressed). The baseline characteristics of the final data for the training and test sets are shown in Table 1. The only significant difference in baseline characteristics between the groups was the number of children (all other variables *p* > 0.05), suggesting that the two groups were not biased by an uneven distribution of the dependent variable.

**Table 1:** Baseline characteristics of the training and test data cohorts.

Variable	Training set (*n* = 2,420)	Testing set (*n* = 1,037)	*p*-value
Gender			
Female	1,295 (53.51)	539 (51.98)	0.428
Male	1,125 (46.49)	498 (48.02)	
Marry			
Unmarried	253 (10.45)	97 (9.35)	0.357
Married	2,167 (89.55)	940 (90.65)	
Incapacity			
No	1905 (78.72)	805 (77.63)	0.503
Yes	515 (21.28)	232 (22.37)	
High blood pressure			
No	1,531 (63.26)	643 (60.06)	0.507
Yes	889 (36.74)	394 (39.94)	
Major activity			
No	913 (37.73)	404 (38.96)	0.519
Yes	1,507 (62.27)	633 (61.04)	
Light activity			
No	466 (19.26)	174 (16.78)	0.095
Yes	1954 (80.74)	863 (83.22)	
Drinking			
No	1,466 (60.58)	617 (59.50)	0.578
Yes	954 (39.42)	420 (40.50)	
Smoking			
No	1728 (71.40)	744 (71.75)	0.871
Yes	692 (28.60)	293 (28.25)	
Retired			
No	1891 (78.14)	817 (78.79)	0.707
Yes	529 (21.86)	220 (21.21)	
Active			
No	1,089 (45.00)	457 (44.07)	0.641
Yes	1,331 (55.00)	580 (55.93)	
Disability			
No	1765 (72.93)	751 (72.42)	0.788
Yes	655 (27.07)	286 (27.58)	
Pain			
No	1,111 (45.91)	439 (42.33)	0.057
Yes	1,309 (54.09)	598 (57.67)	
Satisfaction with life			
Very dissatisfied	31 (1.28)	16 (1.54)	0.6795
Dissatisfied	162 (6.69)	79 (7.62)	
Neutral	1,492 (61.65)	632 (60.95)	
Satisfied	613 (25.33)	266 (25.65)	
Very satisfied	122 (5.04)	44 (4.24)	
Education level			
Primary school	533 (22.02)	240 (23.14)	0.778
Elementary school	677 (27.98)	285 (27.48)	
Middle school	759 (31.36)	331 (31.92)	
High school or above	451 (18.64)	181 (17.45)	
Exercise			
No	180 (7.44)	60 (5.79)	0.093
Yes	2,240 (92.56)	977 (94.21)	
Pension			
No	319 (13.18)	129 (12.44)	0.589
Yes	2,101 (86.82)	908 (87.56)	
Hip injury			
No	2,402 (99.26)	1,032 (99.52)	0.523
Yes	18 (0.74)	5 (0.48)	
Falls			
No	2036 (84.13)	868 (83.70)	0.791
Yes	384 (15.87)	169 (16.30)	
Age	61 [54, 68]	62 [55, 69]	0.126
Income_total	38,300 [9,700, 75,020]	36,000 [10,100, 73,440]	0.295
Household health care percentage	14,550 [8364.167, 23777.25]	14,740 [8573.2, 24,025]	0.685
Family_size	2 [2, 4]	2 [2, 4]	0.464
Household children	2 [1.75, 3]	2 [2, 3]	0.02
Total consumption amount	400 [0, 4,000]	500 [0, 4,000]	0.491
Sleep	6 [5, 7]	6 [5, 7]	0.578
Total_cognition	13.5 [11.5, 15.5]	13.5 [11.5, 15]	0.727

### 3.2 Feature selection

**Figure 2:**
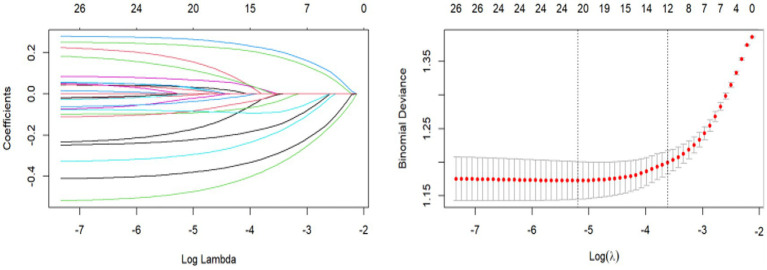
Variable selection by the LASSO regression model. **(A)** Choice of the optimal parameter (*λ*) in the LASSO regression model with logλ as the horizontal coordinate and regression coefficients as the vertical coordinate; **(B)** Plot of λ vs. number of variables with logλ as the bottom horizontal coordinate, binomial deviance as the vertical coordinate, and number of variables as the top horizontal coordinate.

To identify the variables most closely associated with depression, we standardized the training set to eliminate the effect on independent variables due to different units of measurement. With depression as the dependent variable, we used LASSO regression to prevent overfitting by variable coefficient compression and address multicollinearity (see Figure 2). We used 10-fold cross-validation to determine the optimal penalty parameter *λ*. The predictive performance of the model was assessed by calculating the binomial bias of the test data. The R package automatically generates two values of λ, one that minimizes the binomial bias and another that maximizes λ within 1 standard deviation of the minimum bias. We used the latter as it provided a stricter penalty, further reducing the number of independent variables (see Figure 2). The final variables screened by LASSO were as follows: life satisfaction, cognitive ability, pain, sleep, disability, alcohol consumption, education level, total income, gender, retirement status, number of children, and hip fracture. Subsequently, these variables were used as predictors to construct the machine learning model.

### 3.3 Model evaluation and comparison

**Figure 3:**
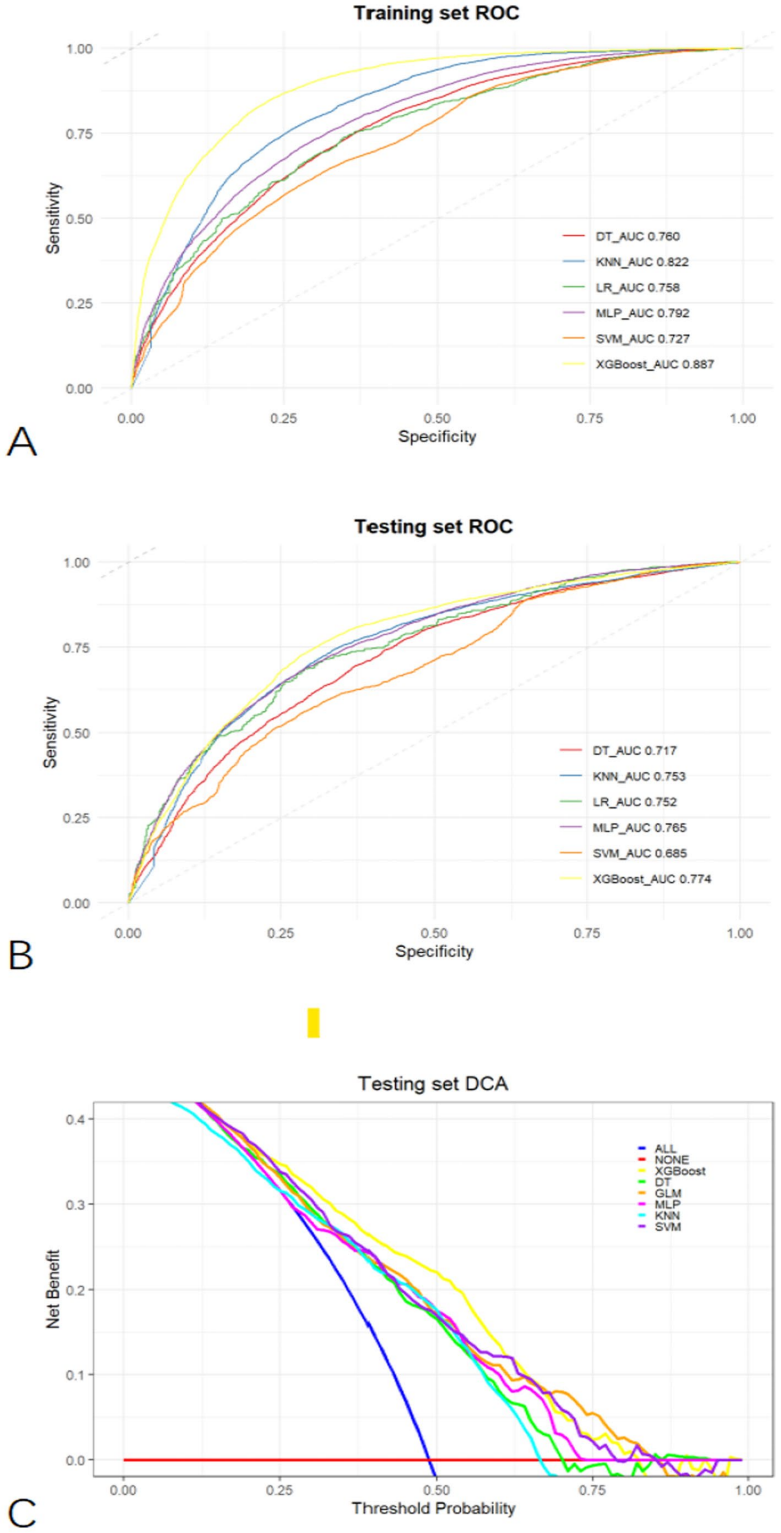
Comprehensive evaluation of machine learning models. **(A)** ROC and AUC of the training set; **(B)** ROC and AUC of the testing set; **(C)** In the testing set, the ALL curve represents the benefit rates for all cases with intervention, while the NONE curve represents the benefit rates for all cases without intervention. The remaining curves denote various models.

Based on the LASSO feature selection results, we constructed predictive models from the training set using several widely used machine learning algorithms, namely LR, KNN, DT, MLP, SVM, and XGBoost. During the modelling process, we repeated five rounds of 10-fold cross-validation and grid search parameter optimization to ensure that the model was not over-fitted and had good generalization ability. The grid search was used to optimize the model’s hyperparameters by exhaustively searching through a specified parameter space. Key parameters, such as the number of estimators, learning rate, max depth (for tree-based models), and regularization terms, were tuned across multiple values. For example, in the XGBoost model, we tested multiple learning_rate and max_depth values of 0.01, 0.05, 0.1, 0.2, and 3, 6, 9, and 12, respectively. in this way, we were able to select the most appropriate hyperparameter while controlling model complexity and avoiding overfitting combinations, thus improving the predictive performance of the model. At the same time, we also tuned the parameters n_estimators, subsample, and colsample_bytree to ensure that the training of each tree maximizes the contribution while preventing overfitting of the data. In the SVM model, we focused on optimizing the penalty parameter (C) and kernel type (kernel). Specifically, we adjusted the values of C (0.1, 1, 10, 100) to balance the model’s tolerance to errors in the training data, and chose appropriate kernel functions, such as linear and rbf, based on the nonlinear characteristics of the data. The tuning process of these hyper-parameters helped us to find the optimal parameter combinations for the SVM model, which improved the model’s generalization ability and avoided the phenomenon of overfitting. The predictive models were evaluated using subject work characteristics (ROC) curves, accuracy, sensitivity, specificity, and F1 scores. The optimal cut-off point was determined by maximizing the Youden index (i.e., sensitivity + specificity − 1) on the ROC curves in the training set prior to evaluation. The XGBoost model had the highest AUC on both the training and test sets (Figures 3A,B) and outperformed other models in all performance metrics except the specificity metric. Full details about the specific model parameters using different algorithms are presented in Table 2. Although a higher AUC represents higher model prediction accuracy, it is not sufficient to assess the practical value of the model. Thus, to compare the actual utility of the different models, we plotted curves on the test set using DCA. The DCA results (Figure 3C) show that XGBoost has the highest net benefit over most threshold ranges. Taken together, these results indicate that XGBoost is the best model.

**Table 2:** Evaluation of the performance of the six algorithms.

Algorithm	Data set	Threshold	AUC	Accuracy	Sensitivity	Specificity	F1
XGBoost	Train	0.506	0.887	0.810	0.848	0.772	0.809
XGBoost	Test	0.483	0.774	0.722	0.757	0.687	0.720
DT	Train	0.443	0.760	0.692	0.777	0.607	0.682
DT	Test	0.430	0.717	0.662	0.740	0.584	0.653
LR	Train	0.454	0.758	0.695	0.738	0.651	0.692
LR	Test	0.479	0.752	0.699	0.686	0.711	0.698
MLP	Train	0.493	0.792	0.716	0.727	0.704	0.715
MLP	Test	0.498	0.765	0.699	0.690	0.709	0.699
KNN	Train	0.562	0.822	0.749	0.767	0.731	0.749
KNN	Test	0.485	0.753	0.703	0.743	0.663	0.701
SVM	Train	0.532	0.727	0.661	0.644	0.677	0.660
SVM	Test	0.547	0.685	0.637	0.560	0.713	0.627

### 3.4 Interpretation of the models

**Figure 4:**
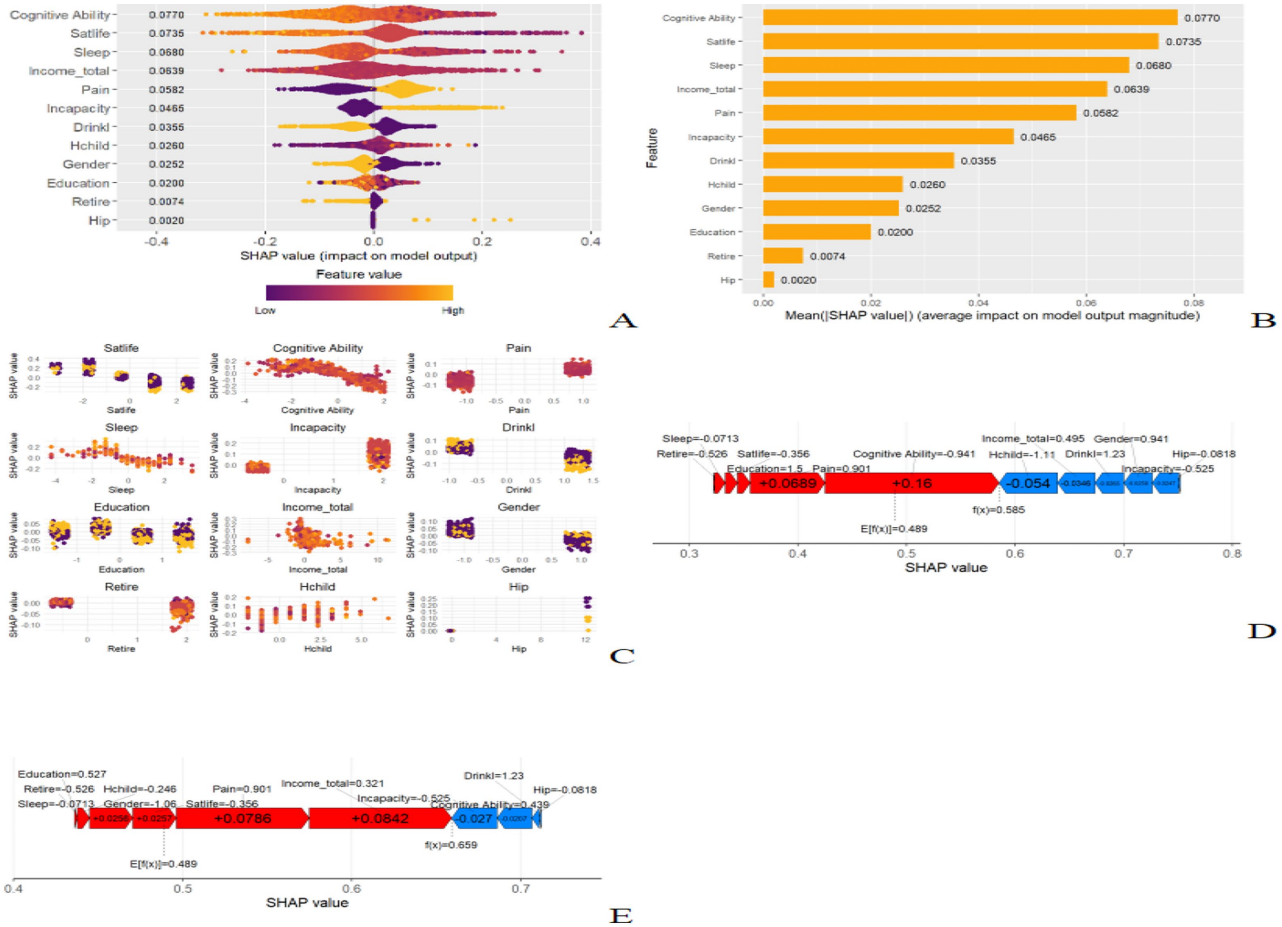
SHAP interprets the model. **(A)** All samples and features are illustrated, with each row representing a feature and x-axis representing the SHAP value. The yellow dots represent higher feature values, while the purple dots represent lower feature values. **(B)** Ranking of variable importance based on the average value. **(C)** The SHAP dependence plot of the XGBoost model. **(D)** SHAP predictions for no disability samples. Arrow direction and color: Blue arrows indicate that the trait contributes negatively to the prediction of depression risk, and increasing the value of the trait decreases depression risk. Red arrows indicate that the feature has a positive contribution to the prediction of depression risk, and increasing the value of the feature will increase depression risk. **(E)** SHAP predictions for samples with depression. Arrow length: the length of the arrow reflects the amount of influence the feature has on the prediction results. Longer arrows indicate that the feature contributes more to the final prediction result, while shorter arrows indicate that the feature has less influence. The value next to each arrow is the SHAP value of the feature, indicating the specific contribution of the feature to the model’s prediction results. Positive values indicate that the feature increased the prediction, while negative values indicate that the feature decreased the prediction. The SHAP value is a central tool for quantifying the impact of a feature, and helps us understand the contribution of each feature in the model.

To better understand the relationship between the best performing XGBoost model and the data, we used SHAP to provide an intuitive illustration of how these variables affect the probability of depression. Figure 4A illustrates the 12 assessed risk factors through the SHAP values. The SHAP values, located on the x-axis, are a unity index that identifies how a feature affects the results of the model. Within each significant feature row, the participant’s attribution of the outcome is plotted with purple and yellow colored dots indicating high and low risk values, respectively. Figure 4B shows the important features in the model, with the ranking of features on the y-axis indicating the importance of the predictive model. The results show a high correlation between cognitive ability, life satisfaction, sleep quality, income level, and depressive symptoms in healthy older adults. The SHAP dependency plot (Figure 4C) can also be used to understand how individual features affect the output of the XGBoost prediction model. To demonstrate the interpretability of the model, we provide two typical examples: one predicting no depression (Figure 4D) and one predicting depression (Figure 4E).

## 4 Discussion

In this study, we developed a predictive model to estimate the risk of depression over 2 years in healthy middle-aged and older adults (≥ 45 years) in China. To achieve this, we employed several machine learning algorithms and used the LASSO method for feature selection. After evaluating six machine learning algorithms, we identified 12 important features to develop and validate the model. Among all models tested, the XGBoost model performed the best in terms of predictive performance. Next, by analyzing the model using SHAP, we identified multiple significant influences including cognitive ability, life satisfaction, sleep quality, income level, and age. Finally, we demonstrated how these features affect the model’s depression prediction ability.

From the perspective of influencing factors, feature selection is crucial for developing predictive models (33). The LASSO algorithm used in this study identified 12 of 26 variables as significant. One identified predictor of depression is life satisfaction. Prior research has shown that individuals with low life satisfaction are more likely to experience depressive symptoms. In a study involving 2,000 older adults, the incidence of depression was twice as high among those with low life satisfaction scores than those with high scores (34). Individuals with low life satisfaction may feel that their lives lack meaning and fulfilment, which in turn triggers depression. Koivumaa-Honkanen et al. reported a significant negative correlation between life satisfaction and depression, finding that individuals with low life satisfaction were approximately 30% more likely to report depressive symptoms (35). These findings suggest that life satisfaction is an important indicator of mental health, especially in older adult individuals. Notably, low life satisfaction is often associated with a lack of social support and financial strain (36). As life satisfaction is closely related to well-being, and low well-being can reduce an individual’s ability to cope with challenges (37). Cognitive decline is also strongly associated with depressive symptoms. Geda et al. found that cognitive dysfunction — particularly deficits in memory and attention — was directly related to the development of depressive symptoms (38). Another study of cognitive function found that the risk of depression increased by approximately 20% for each standard deviation of cognitive decline (39). Richard-Devantoy et al. found that cognitive dysfunction affects an individual’s quality of life and significantly increases the risk of depression. A decline in cognitive ability may lead to individuals experiencing more difficulties in their daily lives and increased feelings of helplessness and social isolation, ultimately increasing the risk of depression (40). This finding emphasizes the key role of cognitive ability in regulating mood and preventing depression. Pain, which indicates chronic physical discomfort, and depressive symptoms often go hand in hand. Approximately 65% of patients with chronic pain also report depressive symptoms (41). Persistent pain can impair one’s ability to regulate their mood, leading to decreased quality of life and increased depression risk. Bair et al. found that the intensity of pain was positively correlated with depression severity, with each unit increase in pain increasing depressive symptom scores by 0.5 points (42). A longitudinal study by Gerrits et al. found that persistent pain significantly increased the prevalence of depression, especially among middle-aged and older individuals, and that pain management plays a key role in the prevention and treatment of depression (43). Studies have also demonstrated a complex interaction between sleep quality and depression. For example, chronic insomnia is considered an independent risk factor for depression. In a longitudinal study, individuals with persistent insomnia were nearly three times more likely to be depressed over a 2 year period (44). Furthermore, Franzen et al. found that poor sleep quality can lead to mood disorders, poor concentration, and fatigue, all of which may exacerbate depressive symptoms (45). A meta-analysis by Baglioni found that improving sleep quality not only improves the quality of life but also reduces the incidence of depression. They showed that each improvement in sleep quality significantly reduced the risk of depression, and this relationship was particularly important among older adults (46). Disability significantly impacts an individual’s ability to perform daily activities and is an important risk factor for depression. According to a study by Verbrugge and Jette, the incidence of depression in disabled individuals was 1.5 times higher than that in non-disabled individuals (47). Reduced social participation and self-care due to disability can increase feelings of loneliness and helplessness, increasing the risk of depression. Prince et al. found that disabled individuals — particularly older adult individuals — commonly face a higher risk of depression due to limitations in their daily lives, and that supporting disabled individuals to participate in social activities and providing appropriate assistance can help reduce depression risk (48). In addition to these five priority factors, other factors are associated with depression. There is a clear association between excessive alcohol consumption and depression, with alcohol abuse potentially leading to increased depressive symptoms (49). Lower education levels may also lead to an increased risk of depression, as education affects an individual’s ability to access resources and social support (50). Low income is also strongly associated with an increased risk of depression (51). Studies examining gender have shown that women are more likely to suffer from depression than men, which may relate to differences in biology or social roles (52). Retirement may also increase the risk of depression, as retirement may lead to loss of roles and reduced social participation (53). Having fewer children may also increase the risk of depression, potentially due to lower family support (54). Finally, physical injuries, such as hip fractures, are important risk factors for depression as they may lead to long-term physical and psychological challenges (55). Collectively, these factors play important roles in the emotional state and mental health of older adults. By better understanding the correlation between these factors and depression, we can more effectively provide early intervention and personalized treatment in clinical practice.

To construct the model, we used a variety of machine learning algorithms to address the challenges of predicting depression risk. Although traditional methods such as discriminant analysis and multivariate logistic regression are easy to use and interpret, they perform poorly when dealing with complex and non-linear data relationships (56–59). Machine learning models, especially non-linear algorithms such as XGBoost, excel in handling complex data relationships. Not only do these models offer improved prediction accuracy, but they can also provide a deeper understanding of complex relationships in data by identifying key influences through feature importance ranking (60–63). Data imbalance is an important issue in model construction. In the data used to predict depression, there are usually more healthy individuals than depressed individuals. Such imbalance can cause the model to be biased towards healthy individuals and thus perform poorly at identifying depressed individuals. To overcome this issue, we employ SMOTE to balance the proportion of classes by generating synthetic samples, thus improving the model’s ability to recognize minority classes (64, 65). Model personalization and interpretability are also important for constructing depression prediction models. As depression may be driven by different factors in different individuals, the use of personalized models can tailor prediction scenarios based on individual characteristics and historical data to enhance prediction accuracy. To enhance the interpretability of the model, we use explanatory techniques such as SHAP values. These techniques reveal the specific contribution of each feature to the prediction results and help identify key risk factors, thus providing a transparent model decision-making process (66, 67). SHAP values reveal how characteristics such as life satisfaction, social support, and cognitive functioning affect depression risk, providing clinicians with practical recommendations for intervention. Overfitting is another important issue in model evaluation. During modelling, some models perform very well on the training set and achieve high accuracy on test data, even when methods such as cross-validation are used. However, this may be due to overfitting of the model on a specific dataset, resulting in insufficient generalization when new data are used (68). To overcome this problem, we use several techniques during model development to reduce the risk of overfitting. First, we aimed to simulate a real model evaluation environment by separating a portion of the dataset for external validation and parameter tuning in the absence of completely independent external validation data. Before feature selection and normalization, we separated a portion of the data for independent testing to ensure that the model maintained a good performance on different datasets. This approach enabled us to achieve better control of the overfitting problem, thus improving the model’s applicability in different populations. Secondly, we used regularization techniques, namely LASSO regression, to prevent model overfitting. Regularization reduces the complexity of the model by imposing penalties on the model parameters, which helps avoid overfitting the training data when dealing with multivariate and complex data. This technique makes the model more robust and able to perform stably on different datasets. We also used multiple cross-validation and hyper-parameter optimization techniques during the model construction process. Cross-validation can effectively evaluate the performance of a model and select the optimal model parameters to avoid bias caused by the training data. Using hyper-parameter optimization methods such as grid search, we were able to systematically explore different combinations of model parameters and identify the optimal parameter settings to enhance the predictive performance and generalization ability of the model.

Depression risk prediction models have a wide range of clinical applications. The practical value of a model depends on its predictive performance as well as its practicality and interpretability (69). Although machine learning models excel in terms of accuracy, their complex internal structure often makes them difficult for clinicians to apply (70). By incorporating explanatory tools such as SHAP values, we aimed to make the model’s decision-making process transparent, enabling clinicians to understand the logic behind the predictions and increasing trust and acceptance of the model (71). Such transparency is essential to facilitate the use of such models in clinical practice (72). Individualized intervention is an important goal of models in clinical application. As patients with depression may present with different symptoms due to various factors, personalized risk prediction can provide clinicians with tailored recommendations for intervention (73). For example, by analyzing the importance of a patient’s characteristics, clinicians could offer psychological support and social resources for patients with low life satisfaction, cognitive training for patients with cognitive decline, and sleep management programs for patients with insomnia problems (74). Personalized interventions can improve the relevance and effectiveness of treatment, ultimately helping patients better manage their depressive symptoms (75). Dynamic adaptability is another key factor of models for clinical application. As patients’ conditions and living circumstances may change over time, models that can be updated in real time will provide more accurate predictions (76). Future development of dynamic prediction models that adapt to changes in a patient’s status, in combination with time series analysis techniques, can help improve the accuracy and clinical utility of predictions (77).

In conclusion, the depression risk prediction model constructed in this study shows good performance and application potential at multiple levels. By addressing issues related to data imbalance, feature selection, and overfitting, and by enhancing the personalization and interpretability of the model, we provide strong support for early diagnosis and personalized intervention of depression (78). Future studies should continue to explore dynamic model development and clinical application integration to further improve the validity and operability of predicting depression risk (79).

## 5 Limitations

The predictive model for depression risk constructed in this study is subject to several limitations. First, the definitions and diagnostic criteria for depression may vary across cultures and healthcare settings, and there are no uniform criteria to define depressive states. Although we used standardized scales and diagnostic tools to assess depression, they may not fully capture the complexity and diversity of the condition, affecting the generalization ability of the model. Second, although the data were derived from a nationwide survey, the data may not adequately cover the diversity of the population across regions, cultures, and socioeconomic contexts. This may limit the applicability and accuracy of the model across regions and populations, especially when the manifestations and influences of depression vary culturally and geographically. Third, the self-reported data used in the study may be subject to recall bias and social desirability effects. Participants may experience memory errors or social pressures when reporting their psychological states and life events, which could lead to underestimation of the effects of key psychosocial factors. Thus, the models may not reflect true depression risk due to data quality and reliability issues. In addition, the variables included in the models were limited by the structure of the questionnaire. Treating certain variables as categorical or continuous, as well as the criteria used to divide the variables, may affect model performance and interpretation of the results. Despite the use of feature selection techniques such as LASSO, the etiology of depression is complex and potentially important factors may be missing from the model. Fourth, the variables and data time points that the model relies on may not reflect the dynamic process of depression. The development of depression is a complex, long-term process that may be affected by unexpected life events, social changes, and other factors that are not adequately reflected in static data. The model in this study is mainly based on the CHARLS dataset, which is derived from the older adult population in China, and thus its ability to generalize to different cultural and regional contexts has not been fully validated. Factors such as socioeconomic structures, health behavior patterns, and healthcare systems in different countries or regions may differ from those in China, which may lead to limitations in the applicability and accuracy of the model in other cultural or geographic contexts. Due to the lack of external validation data, the current study was unable to comprehensively assess the performance of the model on a global scale or in other cultural contexts. Therefore, future research should focus on collecting and analyzing data from different regions to assess the cross-cultural and cross-regional validity of the model to ensure its generalizability and robustness. Finally, although the data and results of this study are somewhat generalizable, they may not be applicable to certain subgroups or settings. Specifically, the predictive performance and applicability of the model should be reassessed and adjusted for populations that differ significantly from the study sample. Despite these limitations, this study provides valuable insights for identifying risk factors and modelling depression, offering a scientific basis for the development of clinical interventions and prevention strategies. Future research could optimize the model and improve its applicability and validity in different populations.

## 6 Directions for future research

There are several key areas for depression risk prediction models that deserve further exploration and development. Firstly, the diversity and breadth of data are fundamental for improving the ability of models to generalize (80). In cross-cultural and multi-center data collection, cultural differences between different geographical regions and populations need to be considered to enhance the applicability of models in different contexts (81). The integration of multimodal data (e.g., genetic information, neuroimaging data, socioeconomic factors, etc.) could provide a more comprehensive view of the pathogenesis of depression, in turn enhancing the accuracy and interpretability of model predictions (82, 83). In terms of model development, adaptive and deep learning techniques offer new possibilities for model optimization (84) Adaptive learning models can be optimized through real-time updating and respond to dynamically changing patient data using updated parameters (85), while deep learning techniques can capture complex nonlinear relationships that are difficult to identify using traditional models (86). Although interpretability is key to a model’s success, model interpretability remains a challenge. Although tools such as SHAP reveal the internal mechanisms of models to some extent, the development of more transparent and intuitive interpretation methods will help clinicians better understand and apply models (87).

Finally, personalized intervention strategies should be the focus of depression risk prediction. Such models can identify high-risk individuals and tailor treatment plans, such as psychological counselling, pharmacological interventions, and social support (88). Future research should aim to combine predictive models with real-time monitoring technology to adjust and optimize interventions in a timely manner by acquiring and analyzing continuous data, ultimately improving treatment outcomes and patients’ quality of life (89).

## 7 Conclusion

In this study, we successfully applied machine learning methods to predict the risk of depression in older Chinese adults over a 2 year period, finding that the XGBoost model performed particularly well at this task. To address the “black box” problem, we used SHAP for model interpretation. In addition to clarifying the importance of each feature in the model, SHAP revealed how these features specifically affect depression risk prediction. This interpretive power enhances the transparency and credibility of the models and opens new possibilities for early identification and intervention for older adults at risk of depression. Future research could further optimize these models and incorporate more personalized data to improve the accuracy of predictions and the effectiveness of interventions.

## Data Availability

Publicly available datasets were analyzed in this study. This data can be found here: http://charls.pku.edu.cn/.
